# Effects of Non-Invasive Neuromodulation of the Vagus Nerve for the Management of Cluster Headache: A Systematic Review

**DOI:** 10.3390/jcm12196315

**Published:** 2023-09-30

**Authors:** David Fernández-Hernando, Cristian Justribó Manion, Juan A. Pareja, Francisco J. García-Esteo, Juan A. Mesa-Jiménez

**Affiliations:** 1 Universidad San Pablo—CEU, CEU Universities, Urbanización Montepríncipe, Boadilla del Monte, 28660 Madrid, Spain; 2 Universidad Abat Oliba—CEU, CEU Universities, 08022 Barcelona, Spain; cristian@cmtosteopatia.com; 3 Department of Neurology, Hospital Quirón Pozuelo, 28223 Madrid, Spain; japarejagrande@gmail.com; 4 Facultad de Medicina, Universidad San Pablo CEU, 28925 Alcorcón, Spain; franciscojavier.garciaesteo@ceu.es; 5 Department Physical Therapy, Faculty of Medicine, University of San-Pablo CEU, Campus Montepríncipe, Urbanización Montepríncipe, 28925 Alcorcón, Spain; jmesaj@ceu.es; 6 Research Laboratory INCRAFT—Interdisciplinary Craniofacial Pain Therapy, 28049 Madrid, Spain

**Keywords:** systematic review, cluster headache, headache, non-invasive neuromodulation, vagus nerve, related disability, auricular transcutaneous vagus nerve stimulation

## Abstract

Background: Cluster headache (CH) is a type of headache that has a global prevalence of 0.5–3/1000 people, provokes severe, strictly unilateral pain through the first branch of the trigeminal nerve, and is associated with observable autonomous responses. CH provokes intense pain and decreases quality of life. Objective: In this study, we aimed to carry out a systematic review of the effectiveness of non-invasive neuromodulation of the vagus nerve in patients with cluster headaches, which was registered on PROSPERO No. CRD42021265126. Methods: Six databases were used from their date of inception to February 2023 to obtain studies with the group intervention of non-invasive neuromodulation of the vagus nerve for cluster headache, with outcomes based on pain attacks, duration, and disabilities. Data on the subjects, group intervention, main outcomes, and results were collected by two authors. Results: The search provided 1003 articles, with three clinical trials being eligible for inclusion in the review. The methodological quality scores ranged from 6 to 8 points (mean: 7.3, SD: 0.8) out of a maximum of 10 points. The post-treatment results showed some positive effects using n-VNS as a treatment for cluster headache, more specifically regarding cervical neuromodulation of the vagus nerve. Conclusions: The systematic review found moderate-to-high-quality evidence supporting that n-VNS and cervical n-VNS may have some positive effects at the end of the treatment being effective to relieve the frequency and intensity of cluster headaches. The poor quantity of studies available and the lack of homogeneity in the study protocols did not allow the pooling of data for a meta-analysis.

## 1. Introduction

Cluster headache (CH) is a type of primary headache that affects around 0.5–3/1000 people worldwide, but there are some differences depending on the geographical area [1]. It is more prevalent in males than in females and affects mainly those under 50 years of age and with a family history of the illness, which encompasses around 6.27% of the global population (95% CI: 4.65–8.40%) [2]. The main characteristics are severe, unilateral pain that is associated with autonomous features, causes disability, and significantly reduces quality of life [3,4]. The main symptoms are unilateral pain attacks related to ipsilateral conjunctival injection, lacrimation, nasal congestion, rhinorrhea, forehead and facial sweating, miosis, ptosis, and/or eyelid oedema, and restlessness or agitation. The attacks can last between 15 min and 3 h and occur every other day to eight times a day, which follows the circadian rhythm [5]. The most common type is episodic CH (85%), with different phases lasting at least three months and remission periods [6,7]. Chronic CH is shorter but has a burning feeling, which makes it more painful.

The current etiology of cluster headache is unclear; it could be an explanation that may provide evidence regarding the neurological pathways through the cervical and trigeminal afferents [7,8,9,10]. There are mediators of neurogenic inflammation linked to that type of headache and an altered response of the immune system associated with central sensitization [8]. There are publications related to headache pain with abnormalities in the brainstem nuclei and cortical superior areas of the brain; on the other hand, headaches could amplify the processing within the peripheral and central trigemino-vascular afferents and the possible connection between them [8,9].

According to the most current literature, one of the possible explanations for this phenomenon in relation to cluster headache with a neuro-physiological relationship is the interconnection between the nucleus of the solitary tract, the nucleus ambiguous (NA), the trigeminal spinal nucleus (TSN), and the dorsal motor nucleus of the vagus nerve. Therefore, the central nervous system is connected with the TSN through the external cuneate nucleus. In turn, the NTS spreads to other neuro-anatomical areas, like the external cuneate nucleus, and continues to the solitary tract. In addition, some fibers of the NTS end at the dorsal or ventral part of the dorsal raphe nuclei, the locus coeruleus, the dorsal medial medulla, the lateral paragigantocellular reticular nucleus, and the medial and lateral parabrachial nuclei. Furthermore, the NTS has connections with other cranial nerves, such as V, VII, X, and XII, and projects back to the dorsal motor nucleus of the vagus nerve (DMX). Furthermore, based on non-invasive neuromodulation of the vagus nerve (n-VNS) in primary headache disorders, the current evidence regarding the trigeminovagal complex in the brainstem could further explain the relationship between the peripheral branch of the vagus nerve and superior brain areas, which are linked to other areas. Other publications using fMRI support the relationship between the NTS and the spinal trigeminal nucleus (STN) with n-VNS as a nerve activation of the auricular branch areas of the vagus nerve. In addition, a cervical application of n-VNS is capable of activating A and B fibers, but not C fibers, as a treatment for this primary headache because n-VNS interacts at the levels of extracellular glutamate in the caudal part of the TSN and NTS, and those brain areas are deactivated because of the n-VNS and the anti-inflammatory effect of this type of neuromodulation of the vagus nerve [11,12].

One of the latest clinical guidelines has shown that the use of pharmacology for the treatment of headaches has adverse effects, such as the use of oral corticosteroids and corticosteroid injections, prophylactic therapies, and other pharmacological therapies, and there is no clear evidence regarding the optimum quantities or the ideal areas for the injections to be applied. This is one of the lowest grades of recommendation, according to the clinical guidelines [13].

Neuromodulation therapies have demonstrated the highest degree of recommendation as a treatment option. These include invasive and non-invasive neuromodulation, with invasive neuromodulation being more effective but costly and having high adverse effects for patients in comparison to non-invasive neuromodulation, which is less costly and safer with fewer adverse effects [13].

In non-invasive neuromodulation of the vagus nerve, there are two areas of application of the therapy: one is in the auricular area, and the other is in the cervical area, located more specifically in the sternocleidomastoid muscle. The first stimulates the auricular branchial arch of the vagus nerve, which contains autonomic afferents, while the second stimulates the lateral side of the neck, where the sternocleidomastoid is located [14]. One of the main differences is that the non-invasive modality does not require surgical intervention or anesthesia of any kind, making it safer and without adverse or less severe adverse effects [15,16,17,18,19,20]. It is used in clinical practice for treating different types of neurological pathologies or disorders like chronic migraine, headache, depression, epilepsy, or pain [15,16,17,18,19].

In terms of cost, invasive neuromodulation costs between USD 30,000 and USD 50,000 [15,16,17,18]. In recent years, magnetic resonance imaging (MRI) has demonstrated connections between areas of the body, such as the auricular or cervical branch, in relation to areas of the central nervous system [10,11,12,13,14,15,16,17,18,19,20,21,22]. However, previous studies are not homogeneous in terms of the parameters used, such as frequency, intensity, and waveform, and their results. It has been possible to demonstrate neuro-physiological relationships by MRI, although further studies are still needed [18,19,20,21,22,23]. On the other hand, in research on cluster headaches in relation to n-VNS, the evidence shows a relationship between this type of headache and the neurophysiological system linked to the nervous system. Furthermore, cortical spreading depression (CSD) can be reduced using n-VNS because of its anti-inflammatory effects, which are related to the cholinergic anti-inflammatory pathway. Additionally, the positive effects on trigeminocervical pain neurons and allodynia reported in studies on rats could be applied to humans. In addition, n-VNS has shown evidence of its influence over the GABA system and the norepinephrinergic brainstem nucleus that receives vagal input via NTS. These publications have also shown the influence of n-VNS, along with fMRI, in the autonomous nervous system [10,14,23,24,25,26].

In relation to auricular and cervical stimulation, it is worth noting the differences in the parameters used for the areas where each is applied. The latest publications regarding the most used and/or most effective parameters for cluster headache are 25 Hz every 500 μs for the auricular branch. While for the cervical vagus branch, it is 5 kHz every 40 ms, with a maximum of 25 Hz and an amperage of 60 mA and 24 V [26]. In recent years, it has been possible to relate the parasympathetic relationship of the vagus nerve to the brain, such as the locus coeruleus, NTS, and the trigeminal nerve [26,27,28].

Two reviews have been published, one of which summarized invasive and non-invasive neuromodulation and only listed the clinical outcomes. The other review looked at migraine and cluster headache together without making a clear distinction between the two [26,27]. Another systematic review with meta-analysis examined RCTs and observational studies (retrospective and prospective case-control studies) that included invasive and non-invasive techniques of vagus nerve neuromodulation [28].

### Objectives

- Compilation and synthesis of evidence on non-invasive neuromodulation treatment of cluster headaches (auricular and cervical neuromodulation).
- To evaluate if chronic non-invasive neuromodulation treatment is effective for the treatment of cluster headaches.
- To provide, if possible, doses and frequencies that have been shown to be effective.
- To inform future research and clinical practice based on the review findings.

The novelty of this project is that it provides strong evidence regarding the effectiveness of n-NVS for cluster headache since there has been only one publication on the use of n-NVS in the neck. This review follows high standards for including and assessing research studies, such as the PEDRo scale, ROB-2, and GRADE assessment, in comparison with other publications that did not assess all of these criteria and only selected RCTs as representative of high-quality clinical trials.

## 2. Methods

A systematic review following the PRISMA statement [29] was carried out. This review was prospectively registered in PROSPERO (ID number: CDRXXXXX).

### 2.1. Search Strategy

The databases CINAHL, MEDLINE, PUBMED, PEDro, and WoS (Web of Science) were searched by a reviewer up to February 2023. The search is based on the clinical question proposed below, and the study selection must include a group of people who have cluster headache diagnoses who have had treatment with any type of non-invasive neuromodulation of the vagus nerve, including the auricula or neck area, in comparison with a placebo group. The reports that were included were RCTs, non-randomized controlled clinical trials, and pilot studies. The terminology employed was based on MeSH terms and included the following: 

((non-invasive neuromodulation) OR (auricular transcutaneous vagus nerve stimulation) OR (transcutaneous vagus nerve stimulation)) AND ((cluster headache) OR (headache)).

### 2.2. PICO Question

This systematic review was carried out to resolve a concerning question: “Is non-invasive neuromodulation effective for the management of cluster headache?” 

Participants: People with cluster headaches.

Intervention: Administration of the auricular/cervical transcutaneous vagus nerve stimulation technique.

Comparison: All types of placebos and/or side events.

Outcomes: Intensity and frequency of cluster headache attacks were considered the main outcomes.

### 2.3. Study Selection

The inclusion criteria were as follows: (i) participants: studies with people of any age or sex with cluster headaches, whether episodic or chronic; (ii) the use of non-invasive neuromodulation in the auricular or neck area; (iii) comparator: all types of placebo; (iv) outcomes: pain intensity after treatment, mean attack frequency and duration of cluster headache attacks, mean differences in responders and sustained responders, and adverse events; and (v) studies: randomized controlled clinical trials, non-randomized controlled trials, and pilot clinical trials were targeted since these designs are suitable to answer our research question.

The exclusion criteria were as follows: (i) population: studies in which participants did not present a cluster headache; (ii) intervention: the use of surgical interventions in VNS; (iii) comparator: studies with no placebo group; and (iv) studies: systematic reviews, meta-analyses, narrative-reviews, case reports, letters to the editor, conference presentations, cases, and cohort studies.

### 2.4. Data Extraction and Quality Assessment

The data was extracted by two reviewers and collected in an Excel sheet (Version 16.16.27 201012, Microsoft Excel ^®^). All the information collected was sample size, diagnosis, duration of symptoms, intervention group, main variables, and side effects. In cases of a lack of agreement, a third author would decide to reach an agreement.

The methodological assessment of the quality of the studies was conducted by two independent authors. With the use of PEDro and ROB as rating scales, a third author would decide to reach consensus.

The ROB Scale marks a “Yes” when a low risk of bias is present or not detected as high, and a “No” in the step of detecting a high risk of bias. If the study does not provide sufficient information to identify bias, it is marked as “uncertain” following the Cochrane Collaboration’s tool [27,30]. This scale includes the following items: selection bias, detection bias, attrition bias, and reporting bias.

Whereas the PEDro scale is based on 11 items, of which the last ten are marked with “Yes” or “No”. Unlike the ROB scale, the PEDro scale assesses the randomization process, blinding of the lead study author, therapists, participants, and assessors, sample loss, intention to treat, and whether the difference between groups and points of variability are reported [31]. It is scored from 0 to 10. In the event of a discrepancy between the assessors, a third party would be responsible for resolving the discrepancies.

The Oxford scale is a simple scale that assesses scientific quality based on the type of study at five levels: level 1 is systematic reviews, level 2 is randomized controlled studies, level 3 is cohort studies, level 4 is case studies and series, and level 5 is mechanism-based reasoning, which is the lowest one [32].

This systematic review also used the Grading of Recommendations Assessment, Development, and Evaluation (GRADE) approach. The GRADE scale evaluates the existing evidence and then, based on its quality, issues grades of recommendation, including clinical guidelines. It can be classified as high quality, moderate quality, or low quality. The GRADE approach evaluates quality according to four main domains: risk of bias, inconsistency, indirectness, and imprecision. The overall classification of evidence is assessed as either “high”, “moderate”, “low”, or “very low” using the Cochrane risk-of-bias tool [33].

## 3. Results

### 3.1. Study Selection and Characteristics

**Figure 1:**
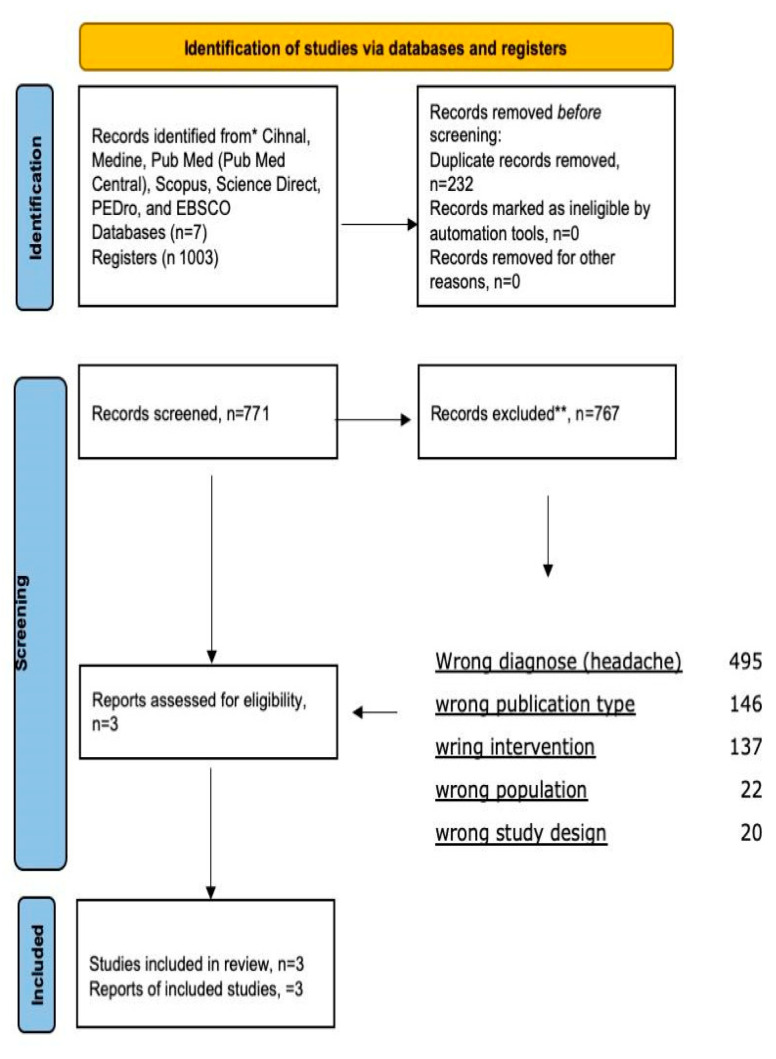
PRISMA flow diagram. * All data bases used for the research. ** All records eliminated based on inclusion & exclusion criteria.

The search provided a total of 1003 articles, including duplicates. The studies that were excluded by screening their title and abstract (*n* = 767) and two hundred and thirty-two (*n* = 232) articles were excluded because they were duplicates of potential eligible studies. Three (*n* = 3) articles were initially included for abstract/full-text review and were then excluded because they focused on other pathologies or areas of application. Finally, only nine articles were included in the systematic review. Figure 1 shows the flow diagram. This systematic review followed the PRSIMA 2020 statement and its available in Table S1.

Table 1 Summary of the studies included in the review [34,35,36]. The total sample size consisted of 332 participants (mean age: 45.7, SD: 11.4; 19.87% women and 80.12% men). The duration of cluster headache-associated symptoms was 82.63 months (SD: 76.14 months), and the frequency of attacks per month was 82.63 min (SD: 76.1) [34,35,36].

### 3.2. Outcomes

We extracted the following outcomes: pain intensity after treatment; mean attack frequency and duration of cluster headache attacks; mean differences in responders and sustained responders; and adverse events [34,35,36].

All outcomes were measured post-treatment, and the follow-ups were weekly based on each study’s design. One of the studies included follow-ups until 8 weeks post-treatment; another study included follow-ups until 48 weeks [34,35,36].

### 3.3. Interventions

For the intervention group, three studies investigated n-VNS, and one study used VNS with an ear implant. For most of these studies, the n-VNS devices were placed in the tract of the vagus nerve; one study did not give more details, another study placed the device on the right side of the neck, and the last study placed the implant near the occipital nerve. 

The parameters of each device in all studies were not very well described, typically a pulse width of 200 mS, a frequency of 5 kHz, and a maximum intensity of 24 V [34]. One study described an n-VNS protocol involving self-administration of three 2-minute prophylactic stimulations (each separated by a period of 5 min) to the right side of the neck (right vagus nerve), and another study used a preventive treatment regimen that occurred twice daily for a total of six stimulations per day with 5000 Hz pulses repeated at a rate of 25 Hz [35]. In the study with the implant, the parameters for stimulation frequency and pulse width were fixed at 60 Hz and 450 μs, respectively, and the intensity was increased in two groups to 30% for one group and the maximum tolerable intensity of 100% for the other group. In the fourth study, the parameters used for nVNS were 1 ms (five sine waves, each lasting 200 microseconds), with such bursts repeated once every 40 milliseconds (25 Hz), generating a 24 V peak voltage and 60 mA peak output current, and the participants could adjust the stimulation amplitude [36].

By contrast, the usual sham at-VNS was a placebo device producing a low-frequency biphasic stimulation that could be perceived as a tingling sensation but with no stimulation to the nerve or provoking muscle contraction, or another therapy like standard of care (SoC) [34,35,36]. 

### 3.4. Methodological Quality, Risk of Bias, and Quality of Evidence

**Figure 2:**
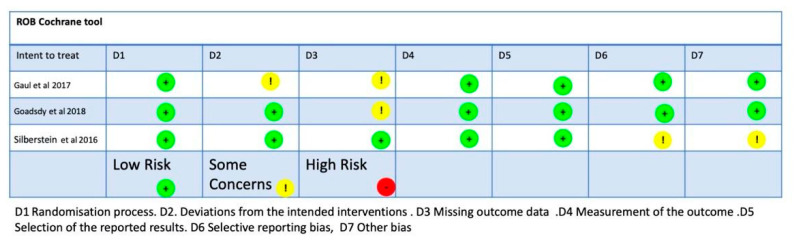
Rob−2 Cochrane tool. D1: randomization process; D2: deviations from intended interventions; D3: missing outcome data; D4: measurement of outcomes; D5: selection of reported results [34,35,36].

All scales to assess the quality of the studies ranged from 6 to 8 points (mean: 7.3, SD: 0.8) out of a maximum of 10 points (Table 2). Two studies [34,36] obtained a high methodological quality score (ε5 points), and one study obtained a low methodological quality score [35]. The ROB-2 Cochrane tool provided a low risk of bias for two studies [34,36], some concerns for one study [35], and a high risk of bias for one study [32] (Figure 2). The graph of the risk of bias shows the following: random sequence generation (D1), 66.6% with a low risk and 33.3% with some concerns; allocation concealment (D2), 66.6% with a low risk and 33.3% with some concerns; blinding of subjects and researchers (D3), 66.6% with a low risk and 33.3% with some concerns; blinding of outcome assessment (D4), 66.6% with a low risk and 33.3% with some concerns; and incomplete outcome data (D5), 66.6% with a low risk and 33.3% with some concerns. According to the Oxford grading of evidence, all studies were marked as level 1 (100%) (Table 3). The GRADE approach obtained the following results: The risk of bias was categorized as low for two studies [35,36] and moderate for one study [34]; in terms of imprecision, all three studies obtained a no; in terms of inconsistency, the three studies might have a low risk; in terms of indirectness, the risk was low/acceptable; and for the last domain, all three studies had a moderate risk of publication bias [34,35,36] (Table 4). 

### 3.5. Summary of Results

Based on the studies included, medium-to-high-quality evidence provided positive results of nVNS in the treatment of CH attacks; nVNS was superior to sham therapy for the treatment of eCH but not for the treatment of cCH in one of the studies, as presented in Table 1. We could extract data on the efficacy, safety, and tolerability of nVNS for the acute treatment of eCH [34]. Meanwhile, another study provided positive results regarding a reduction in chronic cluster headache attack frequency within two weeks after its addition to SoC, showing significantly higher response rates of ≥25%, ≥50%, and ≥75% than SoC alone [35].

The main differences between these two studies are the protocols and the parameters used in each study. This is an important limitation as it prevents the ability to compare the data obtained across studies because of the poor homogeneity in terms of methodology and, thus, further applications of the examined protocols and parameters.

The third study provided enough details regarding nVNS-treated (eCH, *n* = 38; cCH, *n* = 22) and 73 sham-treated (eCH, *n* = 47; cCH, *n* = 26) subjects. A response was achieved in 26.7% of the nVNS-treated subjects and 15.1% of the sham-treated subjects (*p* = 0.1). The response rate was significantly higher with nVNS than with the sham treatment for the eCH cohort (nVNS, 34.2%; sham, 10.6%; *p* = 0.008), but not for the cCH cohort (nVNS, 13.6%; sham, 23.1%; *p* = 0.48). The sustained response rates were significantly higher with nVNS for the eCH cohort (*p* = 0.008) and for the total sample (*p* = 0.04) [36].

### 3.6. Adverse Events

There were no important adverse events reported for most of the studies, with the exception of one study due to the use of the ear implant. There were 59 side effects that happened in 46 participants. Of these, 35 serious side effects in 31 participants (given that one patient had a hardware problem both in the blinded and open phases) were hardware-related, i.e., replacement of empty IPGs or dislocation, failure, or fracture of electrodes or leads. One reliable and important severe adverse was in a patient with multiple vascular risk factors who suffered a right-middle cerebral artery transient ischemic attack 1 month and 15 days after implantation and resumption of anti-thrombotic treatment. This event was considered unrelated to the procedure or the device used by the investigators. No deaths were recorded. The number of serious adverse events was similar in both groups. The third study did not report serious ADEs [34,35,36].

## 4. Discussion

The objective of this systematic review was to ascertain the efficacy of n-VNS and cervical n-VNS in treating cluster headaches. The findings indicate that using n-VNS at 5–25 Hz may have beneficial outcomes post-treatment in reducing the frequency of cluster headache attacks in comparison to the control group. However, due to variations in the methodologies of each study, further research is necessary to establish a standardized protocol with precise parameters for optimal treatment application of n-VNS for patients with CH. Notably, the protocols implemented in each study differed in parameters and follow-up periods, impeding the pooling of data for in-depth analysis and thus precluding a meta-analysis. Further studies of high methodological quality, low bias risk, and similar designs are necessary to analyze the data comprehensively and come to a firm conclusion on the efficacy of n-VNS in the treatment of CH.

This review supports our theory that non-invasive vagus nerve stimulation (n-VNS) is a cost-effective and beneficial treatment option [37]. Compared to current pharmaceutical treatments that are often costly, this alternative approach offers a cost-effective solution [38].

One study on the use of n-VNS in CH measured quality of life using the EQ-5D-3L index. It was the same study that provided evidence of a novel device for n-VNS to treat and improve the quality of life of migraineurs. In fact, the use of this device showed fewer adverse events compared to the use of traditional devices or even surgical implants of VNS as a treatment [39].

The most recent review regarding n-VNS in cluster headaches and migraines was not so clear in the quality of the studies included and the difference in the types of cluster headaches. In comparison, we made some important differences regarding the selection of studies and the evaluation of methodological quality, risk of bias, and quality of evidence. Our selection of studies was based on the criteria that all studies had to be randomized with a control group to extract strong results with the best evidence. Comparing the previous review and our review, the previous review did not evaluate the quality of the methods used and only distinguished the included studies by application and pathology. Thus, it is challenging to know which studies had reliable results. We performed an analysis on the level of evidence, risk of bias, and methodology based on the Oxford score, ROB-2, and PEDro score. One important point regarding the included studies is the heterogeneity of their results. We extracted the parameters, information on the follow-ups, and the main outcomes of each study, and we determined the risk of bias, the quality of the methodology, and the level of evidence to know which studies should be included. Our review shows the necessity of using similar protocols to enable the pooling of data for a meta-analysis. The studies reported the importance of techniques with low costs to be able to treat as many patients as possible. n-VNS is a remarkable treatment option with fewer or even no adverse side effects reported for the treatment of CH, as well as in other studies for the treatment of tinnitus or chronic migraine. One of the studies that was not included in the systematic review was a previous work by Goabsdy et al. [39]. The reason for the exclusion was that the same study was applied in a non-post hoc analysis. All the evidence in this systematic review was rated as of low or moderate quality according to the GRADE assessment (Table 3). This could be because all RCTs included had to improve their methodological study design and provide their outcomes and data in a clearer manner; only one study provided the sample size calculation, and another study did not provide the ID clinical number registration. Thus, the risks of bias, including publication risk, are potential issues that future studies should take into consideration to improve their quality and to provide strong results and conclusions for further recommendations. In contrast to previous systematic reviews, this is the first review using the GRADE assessment and the PEDro scale at the same time to provide strong evidence regarding the risk of bias and methodological assessment. In contrast, other systematic reviews did not include any of these scales to assess the quality of studies and their methodology [25,26,27].

Several studies have investigated the common pathways of tinnitus, migraine, and CH and the mechanisms by which the n-VNS can effectively treat these conditions. These studies have focused on administering stimuli to the peripheral nervous system (PNS) through the central nervous system (CNS) to upper brain regions, such as the hippocampus, amygdala, anterior cingulate cortex (ACC), and hypothalamus [40,41,42,43].

In certain studies on CH, n-VNS, including cervical application, has demonstrated effects on the peripheral nervous system. These effects extend through the central nervous system and reach upper brain areas, such as the ACC, periaqueductal gray (PAG), prefrontal cortex (PFC), cingulate gyrus, supplementary motor area (SMA), amygdala, and thalamus [40,41,42,43,44]. It is unclear how non-invasive vagus nerve stimulation (n-VNS), which includes auricular and cervical n-VNS when utilizing acupuncture points to treat cluster headache (CH), contributes to the function of peripheral vagus nerve branches and the autonomic nervous system (ANS) with current evidence. Nevertheless, there are favorable results, indicating a need for further research with improved methodology to establish definitive conclusions and elucidate the existing pathways and relationships.

The studies reviewed, including the use of fMRI scans, indicate a potential correlation between the beneficial effects of n-VNS in patients with migraines and the brainstem. This may be associated with the brainstem’s influence, specifically the dorsoposterior insula, low medullary brainstem, medial thalamic, ACC, posterior insula, lower medullary brainstem, and medial thalamic/ACC deactivation, when implementing at-VNS/n-VNS. This study offers preliminary evidence supporting the reported connection between the peripheral branch of the vagus nerve and the upper areas of the brain [45,46]. Neuroanatomical research indicates a connection exists between the NTS and the parabrachial area, locus coeruleus, dorsal raphe, periaqueductal gray, thalamus, amygdala, insula, nucleus accumbens, and bed nucleus of the stria terminalis via the left cymba. Furthermore, stimulating brain regions such as the periaqueductal gray, dorsal raphe, and locus coeruleus have been observed to induce anti-nociception. This, in turn, triggers descending inhibitory pathways to the dorsal horn of the spinal cord [47]. There are similarities in the effects of antidepressant and anticonvulsant drugs in some areas of the brain, such as the amygdala, accumbens, hippocampus, dorsal raphe, and locus coeruleus.

There is evidence to suggest a correlation between upper brainstem regions and acupuncture points based on fMRI studies. Implicated areas of the brain encompass the PAG, ACC, left PCC, insula, limbic/paralimbic regions, and precuneus regions [45]. Furthermore, other pathologies, including low back pain, tinnitus, and CH, provide additional evidence of the connection between the brain and peripheral acupuncture points [46]. The brain areas that are stimulated for low back pain include the PFC (prefrontal cortex), insula, cerebellum, SI (secondary somatosensory cortex), and ACC. Similarly, the respective areas for tinnitus and CH are the right MTG (middle temporal gyrus). As previously mentioned, there exists a correlation between peripheral branch nerves and superior brain areas in terms of altering stimulation via n-VNS through fMRI [45,46,47].

Previous systematic reviews on migraine and CH included neuromodulation and cervical n-VNS [26]. These present systematic reviews concentrate solely on transcranial magnetic stimulation (TMS), non-invasive vagus nerve stimulation (nVNS), non-painful remote electrical stimulation (NRES), external trigeminal nerve stimulation (e-TNS), and transcranial direct current stimulation (tDCS) [27]. All reviews indicate a potential small positive effect for the treatment of migraines, CH, and similar pathologies. The outcomes in two studies were comparable in the measurement of pain intensity, the number of attacks, or their duration. In conclusion, the clinical results demonstrate numerous n-VNS applications available for treating CH, whether it is episodic or chronic. Employing these varied n-VNS applications has positive effects and must be considered as a safe treatment option [26,27]. The only treatment for CH with clear evidence is drug therapy, and the most recent publication comparing children with adolescents reaches the same conclusion as in adults [48]. There is not enough strong evidence to support the use of other treatments, and it is mandatory to conduct more RCTs on at-VNS and other treatment choices to have safe and useful choices for the treatment of this condition.

Evidence has shown us the effects on the variability of the heart rate due to the influence of the vagus nerve. According to a recent publication, there are effects of cardiac variability in relation to the vagus and possible non-invasive therapies for its treatment under the influence of the vagus nerve to be taken into account. Therefore, such variability in cardiac rhythm should be considered a variable to be measured in studies, and in particular for those that use non-invasive therapies such as cranio-sacral therapy with positive effects. As well as non-invasive neuromodulation of the vagus, as observed in this systematic review [49].

### 4.1. Strengths and Limitations

The systematic review’s strengths lie in its rigorous methodological assessment using the GRADE, ROB-2, and Oxford scales. This is crucial as it allows for more precise results that can be utilized in the future for meta-analysis. Another vital consideration is the appropriate registration of clinical trials, as only trials meeting high standards are eligible for publication in reputable journals.

There are limitations to the current review. The small number of available studies, even with the positive effect of reducing pain intensity, increases the risk of bias and thus affects the quality of the evidence. Therefore, no firm conclusion can be drawn on the effectiveness of at-NVS and ear electro-acupuncture in managing cluster headaches.

The small number of trials available for the systematic review, along with inconsistencies in follow-up periods and variations in study protocols, prevented performing a meta-analysis. Similar study protocols, including standard parameters, application times, measurements, and electro-acupuncture points, as well as similar pathologies such as episodic or chronic CH, are necessary.

### 4.2. Recommendations and Future Studies

- Future research should clarify key points about the technique’s effectiveness.
- Studies should meet high research quality standards, such as true randomized controlled trials with adequate control groups.
- Quality assessment should be based on scales like PEDro and GRADE.
- The implementation of high-quality studies could support future meta-analyses.
- The recent CONSORT statement publication reinforces the need for improved research quality.
- Inclusion criteria should be consistent across treatment protocols.

## 5. Conclusions

The present systematic review discovers moderate-to-high-quality evidence backing that n-NVS and VNS may have favorable effects post-treatment in reducing the frequency and intensity of cluster headache. However, due to the small number of RCTs and heterogeneity in study protocols, data pooling for a meta-analysis was not possible.

This systematic review emphasizes the need to enhance study quality with quality assessment scales and ensure study design homogeneity. This is necessary for drawing clear conclusions with a low risk of bias [50].

## Figures and Tables

**Table 1:** Summary of all included studies.

Study	Interventions and Groups	SAMPLE SIZE	Male/Female	Age (Years)	Duration CH	Intervention Duration	Comparison and Outcome Measure	Results	AE	Level of Evidence
Gaul et al. 17 RCT [35]	G1;(nVNS + SoC) G2; SoC alone (Sham)	97	G1; 34 male G2;33 male	G1; 45.4 ± 11.0 G2; 42.3 ± 11.0	G1;95.2 ± 57.7 G2; 103.3 ± 66.8 min	The right side of the neck; twice daily for a total of six stimulations per day; side of the neck (right vagus nerve).	Mean Attack Frequencies, response rates, safety, and tolerability.	G1 vs. G2 mean weekly attack *p* > 0.02 G1 vs. G2 attack frequency (*p* < 0.05). G1 vs. G 2; Response rates ≥25%, ≥50%, and ≥75% from baseline (≥25% and ≥50%, *p* < 0.001; ≥75%, *p* = 0.009).	N.R	1
Goadsby et al., 18 [34]	G1; nVNS G2;Sham device	102	G1; 35 male G2; 38 male	G1;43.9 (10.6) G2; 46.9 (10.6)	G1; 69.9 (68.7) G2; 77.4 (76.9) min	G1: 200 mS, frequency 5 kHz, intensity maximum 24 V. Self-administer three consecutive 120- second stimulations ipsilateral to their CH attack.	CH attacks, pain intensity at onset and at 15 and 30 min after initiation of stimulation; rescue treatment use, number of stimulations used, and adverse events.	CH attacks, nVNS was superior to sham therapy in eCH but not in cCH.	nVNS (18%) and sham (19%)	1
Stephen D. Silberstein [36]	G1; nVNS G2; Sham device	133	G1; 59 male G2; 67 male	G1; 47.1 +- 13.5 G2; 48.6 +- 11.7	G1; 86 +- 119 G2; 64 +- 71 min	G1: 5-kHz, 1 ms (each 200 ms), once every 40 ms (25 Hz), 24-V peak voltage and 60-mA peak output current. G2: 0.1 Hz biphasic signal does not stimulate the vagus nerve. Consecutive 2-minute stimulations to the right side of the neck at the onset of premonitory symptoms or pain.	Pain intensity, attack duration, rescue medication use, AEs, device perceptions, and blinding questionnaire responses for each attack.	Response rates (nVNS, 34.2%; sham, 10.6%; *p* 5.008) but not the cCH cohort (nVNS, 13.6%; sham, 23.1%; *p* 5.48). Sustained response rates were significantly higher with nVNS for the eCH cohort (*p* 5.008) and total population (*p* 5.04).	N.R	1

n-VNS—non-invasive vagus nerve stimulation; AEs—adverse events; N.R.—non-reference; cCH—chronic cluster headache; eCH—episodic cluster headache; SoC—standard of care; RCT—randomized controlled trial.

**Table 2:** Score of randomized clinical trials with the PEDro scale.

	1	2	3	4	5	6	7	8	9	10	Total
Goadsby et al., 2018 [34]	Y	N	Y	Y	N	N	Y	Y	Y	Y	7.0
Silberstein et al., 16 [36]	Y	Y	Y	Y	Y	Y	Y	Y	Y	Y	10.0
Gaul et al., 2017 [35]	Y	N	Y	N	N	N	N	Y	N	Y	4.0

1: Random allocation of participants; 2: Concealed allocation; 3: Similarity between groups at baseline; 4: Participant blinding; 5: Therapist blinding; 6: Assessor blinding; 7: Fewer than 15% dropouts; 8: Intention-to-treat analysis; 9: Between-group statistical comparisons; 10: Point measures and variability data. Y: Yes; N: No.

**Table 3:** Oxford scale.

	Level
Goadsby et al., 2018 [34]	1
Silberstein et al., 16 [36]	1
Gaul et al., 2017 [35]	1

**Table 4:** GRADE.

	Risk of Bias	Imprecision	Inconsistency	Indirectness	Publication Bias
Charly Gaul [35]	Low	No	may be low	low/acceptable	moderate
Peter J. Goadsby [36]	moderate	No	may be low	low/acceptable	moderate
Stephen D. Silberstein [34]	Low	No	may be low	low/acceptable	moderate

## Data Availability

Not applicable.

## Supplementary Materials

The following supporting information can be downloaded at: https://www.mdpi.com/article/10.3390/jcm12196315/s1. This systematic review followed the PRSIMA 2020 statement and its available in Table S1.
